# The molecular mechanism by which saturated lysophosphatidylcholine attenuates the metastatic capacity of melanoma cells

**DOI:** 10.1002/2211-5463.12152

**Published:** 2016-11-24

**Authors:** Thomas Ross, Bastian Jakubzig, Manuel Grundmann, Ulrich Massing, Evi Kostenis, Martin Schlesinger, Gerd Bendas

**Affiliations:** ^1^ Department of Pharmaceutical Chemistry IIUniversity of BonnGermany; ^2^ Department of Pharmaceutical BiologyUniversity of BonnGermany; ^3^ Andreas Hettich GmbH & Co. KGF&E Lifescience ApplicationsFreiburgGermany; ^4^ Faculty of Chemistry & PharmacyUniversity of FreiburgGermany

**Keywords:** cancer, G protein‐coupled receptor, integrin, lysophospholipid, metastasis

## Abstract

Lysophophatidylcholine (LysoPC) is an abundant constituent in human plasma. Patients with malignant cancer diseases have attenuated LysoPC plasma levels, and thus LysoPC has been examined as a metabolic biomarker for cancer prediction. Preclinical studies have shown that solid tumor cells drastically degrade LysoPCs by incorporating their free fatty acids into cell membrane phospholipids. In this way, LysoPC C18:0 reduced the metastatic spread of murine melanoma B16.F10 cells in mice. Although membrane rigidification may have a key role in the attenuation of metastasis, evidence for this has yet to be shown. Therefore, the present study aimed to determine how LysoPC reduces the metastatic capacity of B16.F10 cells. Following cellular preincubation with LysoPC C18:0 at increasing concentrations and lengths of time, cell migration was most significantly attenuated with 450 μm LysoPC C18:0 at 72 h. Biosensor measurements suggest that, despite their abundance in B16.F10 cells, LysoPC‐sensitive G protein‐coupled receptors do not appear to contribute to this effect. Instead, the attenuated migration appears to result from changes in cell membrane properties and their effect on underlying signaling pathways, most likely the formation of focal adhesion complexes. Treatment with 450 μm LysoPC C18:0 activates protein kinase C (PKC)δ to phosphorylate syndecan‐4, accompanied by deactivation of PKCα. Subsequently, focal adhesion complex formation was attenuated, as confirmed by the reduced activity of focal adhesion kinase (FAK). Interestingly, 450 μm LysoPC C18:1 did not affect FAK activity, explaining its lower propensity to affect migration and metastasis. Therefore, membrane rigidification by LysoPC C18:0 appears to prevent the formation of focal adhesion complexes, thus affecting integrin activity as a key for metastatic melanoma spread.

**Abbreviations**
DMEM: Dulbecco's modified Eagle's medium
DMR: dynamic mass redistribution
FA: fatty acid
FAK: focal adhesion kinase
FRAP: fluorescence recovery after photobleaching
GPCR: G protein‐coupled receptor
LysoPC: lysophophatidylcholine
PIP_2_: phosphatidylinositol 4,5‐bisphosphate
PKC: protein kinase C
SDC‐4: syndecan‐4

Lysophosphatidylcholine (LysoPC) is a central component of the phospholipid metabolism, which, for example, is formed by enzymatic activity of phospholipase A2 (PLA2) or lecithin‐cholesterol acyltransferase (LCAT) in terms of phospholipid degradation or turnover in lipoproteins 1. LysoPC most likely represents in an albumin‐bound form in plasma, an accessible and efficient cargo to provide fatty acids (FAs) to tissues and organs in a highly dynamic process of the so‐called Land's cycle 2. Therefore, the level of LysoPC in human plasma is well balanced at approximately 300 μm 3 and thus represents around one‐tenth of the total phospholipid fraction in humans 4. However, patients with malignant tumor diseases display significantly reduced plasma levels of LysoPC, as indicated by numerous clinical findings 5, 6, 7. Therefore, in the course of a metabolic profiling, LysoPCs appear to be promising biomarker candidates for the prediction of cancer diseases or their recurrence. Higher levels of LysoPC C18:0 were shown to relate consistently to lower risks of breast, prostate and colorectal cancer 8.

A potential explanation for this phenomenon of deregulated LysoPC level by cancer has been provided by preclinical findings indicating that solid tumor cell lines massively degrade LysoPC 9. As a result, the tumor cells incorporate the LysoPC‐bound FA into their membrane phospholipids, and increase the cellular fraction of neutral lipids by the formation of lipid droplets. This rapid degradation of LysoPC and subsequent FA membrane incorporation is obviously less dependent on specific LysoPC species because, for example, LysoPC C18:0 and LysoPC C18:1 were equally processed by the tumor cell lines 9. Thereby, even higher than physiological plasma concentrations of LysoPC (e.g. 450 μm) were well tolerated by the tumor cells without any sign of cytotoxicity when a near physiological albumin concentration of 22 g·L^−1^ was added to the media. Nevertheless, tumor cells preincubated with this higher LysoPC concentration were affected with respect to function. Most interestingly and of potential therapeutic relevance, murine B16.F10 melanoma cells were strongly attenuated in their metastatic capacity in an experimental metastasis approach in mice 9. The underlying mechanisms of this anti‐metastatic activity of LysoPC remained elusive. Because LysoPC C18:0 was more potent in reducing the metastatic spread than LysoPC C18:1, membrane rigidification appeared to be responsible. Indeed, fluorescence recovery after photobleaching (FRAP) measurements confirmed that LysoPC C18:0 treated B16.F10 displayed a lower membrane lateral mobility than LysoPC C18:1 treated or untreated cells 9.

Membrane fluidity has been described as a factor driving malignancy of breast cancer cells 10, mainly with respect to affecting adhesion properties. We previously demonstrated that LysoPC C18:0 reduces the α_4_β_1_ integrin (VLA‐4) binding capacity in B16.F10 cells 11, which is of key importance for the metastatic spread of these cells 12. Recently, mechanical stiffness has been evaluated as critical factor inversely dictating the invasiveness in a series of ovarian cancer cells 13. Therefore, membrane rigidification by LysoPC C18:0 would match with a reduced malignancy of treated B16.F10 cells. However, it remains unresolved whether attenuated metastasis by membrane rigidification is mainly a biophysically driven process or concurrently affects intracellular downstream signaling pathways.

Concerning signaling pathways, cellular LysoPC activities have also been associated with the effects of G protein‐coupled receptor (GPCR). GPR4, GPR68 (OGR1) and G2A are GPCRs that have been shown to respond to LysoPC 14, 15, 16. However, the effect of these putative LysoPC receptors on reduced cell adhesion has never been investigated thoroughly. A potential interference at the downstream signaling cascade of GPCRs, such as an activation of RhoA GTPases 17, with the signaling pathway of integrins could explain any effects on attenuated cell adhesion in our recent study 11. However, those assumptions are not corroborated by and stand partly in contrast to the results of other studies on GPCR‐related LysoPC effects. For example, these publications describe the endothelial proliferation and migration 18, 19, a facilitated phagocytosis of apoptotic cells 20 or their complex mechanisms in immune reactions 21. Furthermore, different studies have reported pro‐tumorigenic and metastasis promoting effects of LysoPC or lysophosphatidic acid (LysoPA), which result from LysoPC degradation by autotaxin 22, 23, 24. Furthermore, many aspects of LysoPC and GPCR activity are discussed quite controversially 25, generally raising the question of whether these receptors should be considered as proton‐sensitive receptors.

In the light of this open interpretation, the present study aimed to determine the molecular mechanisms of LysoPC in attenuating the metastatic spread of B16.F10 melanoma cells that has been described recently 9, 11. Functionally, we primary focused on cell migration as an essential step in metastasis 26 because LysoPC has been described to affect the motility of other cells, such as endothelial cells in the process of angiogenesis in terms of atherosclerosis 27, 28 or the migration of dendritic cells in immune reactions 29.

We are able to show that the anti‐metastatic activity of higher LysoPC concentrations results from a mechanically‐triggered process of membrane rigidification affecting the signaling pathway of focal adhesion complex formation, which finally attenuates the integrin activation with consequences for reduced migration. This clearly explains the dependency of LysoPC activity on the degree of saturation of the bound FAs and the concentration of LysoPC. Our data exclude the possibility that the observed effects of LysoPC are mediated by GPCRs and their downstream signaling partners.

## Results

### Cell migration is less affected by low LysoPC C18:0 concentrations, independent of GPCR activation

Because migration is an inevitable step within the metastatic cascade, the anti‐metastatic activities of LysoPCs should potentially be reflected by an attenuated migratory capacity of treated tumor cells. LysoPC has been reported to affect the motility of non‐malignant cells 28, 29, although the concentrations of LysoPC used were comparably low (e.g. 12.5 μm in the latter study) and thus differed strongly from the anti‐metastatic approach described recently (450 μm) 9.

**Figure 1:**
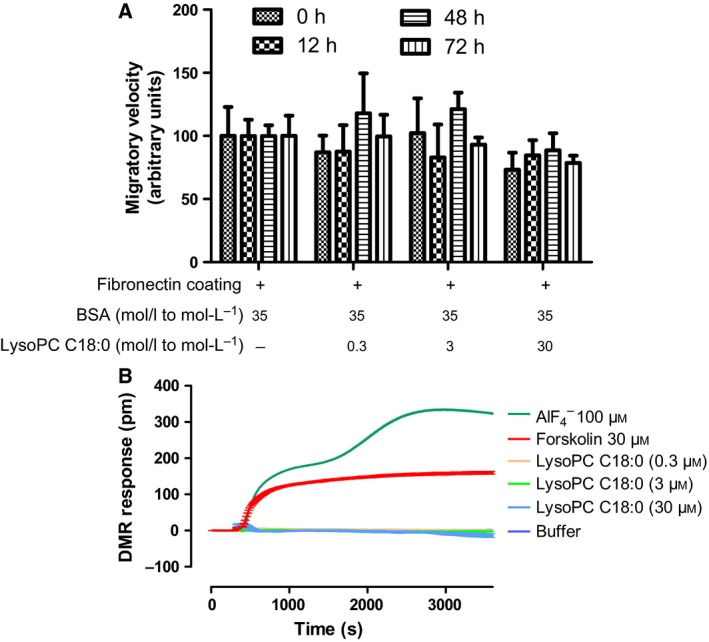
Lower LysoPC concentrations do not significantly affect B16.F10 migration and potential GPCR‐mediated effects of LysoPC can be excluded. (A) Cell migration of B16.F10 cells that have been pretreated with different concentrations of LysoPC C18:0 over various incubation periods in the presence of approximately 35 μm BSA resulting from fetal bovine serum containing media. Migration of nontreated cells was considered as a control (100%). Cell migration was slightly and not significantly affected, although an impact of incubation time on migration was not obvious. (B) Results of an optical biosensor‐based label‐free assay performed with B16.F10 cells. DMR (measured in pm wavelength shift) as a consequence of cellular stimulation is plotted as a function of time considering the SE (*n* = 3–15). Top to bottom: positive controls with the direct G protein activator ${\text{AlF}}_{4}^{-}$ (100 μm) and adenylate cyclase activator forskolin at a concentration of 30 μm induced robust signatures with rapid initial slopes. By contrast, LysoPC C18:0 at concentrations from 0.3 to 30 μm did not initiate a GPCR‐typical activation slope.

To investigate whether low LysoPC C18:0 concentrations affect B16.F10 migration on fibronectin with a dependence on LysoPC preincubation time, we selected a range from 0.3 to 30 μm, each at incubation periods from 0 to 72 h (Fig. 1A). This is below a potential cytotoxic concentration range (IC_50_ of 96 μm LysoPC C18:0 in B16.F10 cells). In general, migration in this two‐dimensional wound healing assay is not affected significantly. There is only a tendency that 30 μm LysoPC slightly diminished the migratory capacities, although the preincubation time appears to be less important. However, the immediate effect of LysoPC without preincubation (0 h data) suggests that receptor‐mediated processes could potentially be involved.

The stimulation of GPCRs by LysoPC appears to be a basis for the multiple biological effects observed in different cellular systems, although various aspects of this activation axis have been discussed quite controversially 25, 30. To investigate whether LysoPC possesses GPCR effects in B16.F10 in relation to the slightly attenuated migration, we initially focused on the three LysoPC receptors, GPR68 (OGR1), GPR4 and G2A, and first confirmed their expression in B16.F10 cells by western blotting. Based on that finding, we investigated the immediate cellular GPCR response to LysoPC exposure in an optical biosensor‐based label‐free assay, which is capable of monitoring holistic cellular changes as dynamic mass redistribution (DMR) in living cells and in real time. This readout has proven particularly suited for capturing GPCR‐mediated cell activation, irrespective of the underlying signaling pathway 31.

LysoPC C18:0 was used in buffer at maximal concentrations of 30 μm, which was reported to be sufficient for activation of putative LysoPC GPCRs 19. In addition, positive controls were performed with the adenylate cyclase activator forskolin at a concentration of 30 μm and the direct G protein activator ${\text{AlF}}_{4}^{-}$ (100 μm), highlighting cell responses that are generally associated with GPCR‐induced signaling 32.

The DMR traces (Fig. 1B) show comparable slopes for the two positive controls, with an even higher final response for ${\text{AlF}}_{4}^{-}$ as a direct G protein activator. LysoPC C18:0, in contrast, completely fails to induce a change in cell morphology. These observations indicate a GPCR‐independent cellular effect of LysoPC, in contrast to bona fide GPCR‐mediated cell responses by ATP acting at P2Y receptors (Fig. S1A).

Similar findings were obtained using the mono‐unsaturated LysoPC species LysoPC C18:1 (Fig. S1A), which promotes only a minor and undefined cell response, reminiscent of the profile in HEK293 cells (Fig. S1B) that do not express GPR68, GPR4 or G2A 33.

### Higher LysoPC concentrations attenuate migration dependent on the degree of saturation of FA

**Figure 2:**
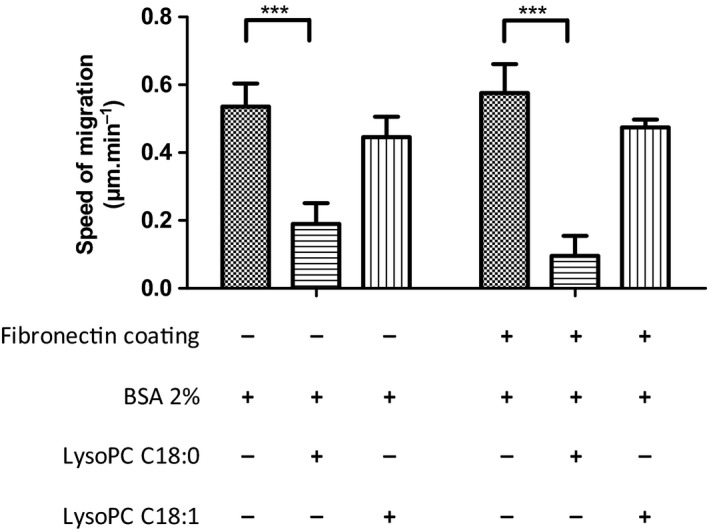
LysoPC at higher concentrations affects the migratory capacity of B16.F10 cells dependent on FA saturation. B16.F10 cells were investigated with respect to their migratory capacity on uncoated or fibronectin‐coated surfaces. Cells were treated with BSA 2%, LysoPC C18:0 or LysoPC C18:1 (both with BSA 2%) at concentrations of 450 μm for 72 h. The speed of migration is indicated (μm·min^−1^). The data show that migration on uncoated surfaces is significantly reduced by LysoPC C18:0 and, to a lesser extent, by LysoPC C18:1. The influence of LysoPC C18:0 with respect to affecting migration is more pronounced on fibronectin‐coated surfaces, suggesting a deregulation of integrin function. By contrast, the effect of LysoPC C18:1 is much lower and comparable in both approaches. Error bar indicates the SD (*n* = 3–9). Asterisks indicate statistical significance: *P < 0.05; **P < 0.01; ***P < 0.001.

Next, we investigated whether higher than physiological LysoPC concentrations affect migration. Thus, we focused on 450 μm, which demonstrated efficient attenuation of the metastatic spread of B16.F10 cells in mice 11. For these experiments, BSA was added to approximate to physiological concentrations. Cell preincubation with 450 μm LysoPC C18:0 significantly attenuated B16.F10 migration. By contrast to the low LysoPC concentrations, preincubation time was decisive and attenuated migration was most pronounced after 72 h. This time range refers to a cell membrane modification by FA incorporation as an underlying mechanism for reduced cell migration. To emphasize the role of FA saturation, B16.F10 cells were preincubated with 450 μm LysoPC C18:0 or LysoPC C18:1 and cell migration was compared on uncoated or fibronectin‐coated surfaces (Fig. 2).

Although 72 h of preincubation of B16.F10 cells with LysoPC C18:0 significantly reduced the cell motility on uncoated surfaces by 65%, the effect of LysoPC C18:1 at the same concentration only induced a reduction of 17% (Fig. 2, left). This graduation in reduced motility is assumed to mainly reflect an attenuated membrane flexibility induced by the LysoPC species.

In the case of the fibronectin‐coated surfaces (Fig. 2, right) the differences between the LysoPC (450 μm, 72 h) versus blank BSA‐treated cells (72 h) are more pronounced. The slightly higher velocity of the BSA‐treated cells in this assay compared to uncoated surfaces indicates a stronger involvement of cellular β1‐integrins for this experiment, where the fully saturated LysoPC C18:0 reduced the migratory velocity significantly by 84% compared to the BSA‐treated B16.F10 cells. Exposure to LysoPC C18:1 led still to a reduction of 18% compared to the BSA control.

The proteoglycan syndecan‐4 (SDC‐4), which also has a receptor function for fibronectin and other extracellular matrix components 34 and, more importantly, a costimulatory binding function for several β1‐integrins 35 is not affected by this LysoPC treatment and displays identical expression levels in treated versus untreated B16.F10 cells (Fig. S2). Furthermore, previous data excluded a downregulation of selected integrins by this type of LysoPC treatment of B16.F10 cells 11.

These findings suggest that integrin activity is affected by LysoPC with this type of treatment, and the degree of saturation appears to have a considerable impact.

**Figure 3:**
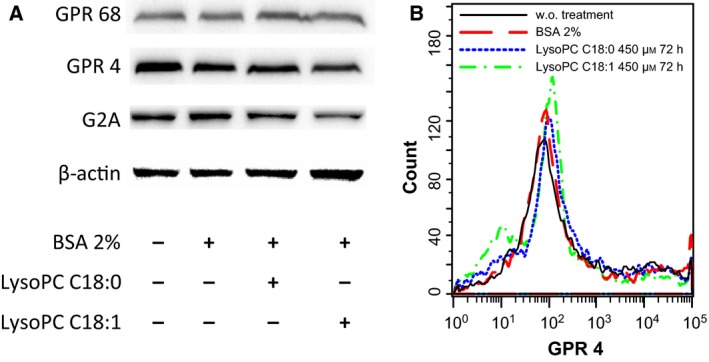
The attenuated migration of B16.F10 cells by higher LysoPC concentrations is not related to GPCR effects. (A) Western blotting of the expression profile of presumed LysoPC‐associated GPCRs, GPR68, GPR4 and G2A, in B16.F10 cells. Prior to cell lysis, the B16.F10 cells were left untreated in DMEM for 72 h, or treated with BSA 2%, or, additionally, with 450 μm LysoPC C18:0 or LysoPC C18:1 for 72 h. The data obtained indicate that this type of LysoPC treatment did not affect the cellular expression profile of the indicated GPCRs. (B) Flow cytometry of GPR4 expression on B16F10 cells confirms that the indicated LysoPC pretreatment does not induce receptor internalization.

Nevertheless, to exclude GPCR effects under these conditions, an additional approach was performed. Because receptor stimulation after 72 h of preincubation appears meaningless and biosensor measurements are affected in the presence of a physiological albumin concentration, an indirect procedure was chosen. Receptor down‐regulation, such as internalization and sequestration, is a characteristic feature of repeated or continuous GPCR stimulation. As indicated before, B16.F10 cells showed significant levels of GPR68, GPR4 and G2A, although 72 h of LysoPC preincubation resulted in no change in expression, neither with LysoPC C18:1 not with LysoPC C18:0 at 450 μm (Fig. 3A). Furthermore, flow cytometry confirms that cells do not respond to LysoPC preincubation by receptor internalization, as shown for GPR4 (Fig. 3B). These data suggest that the receptors in question, although present on B16.F10 cells, might not be targeted by the LysoPC species used in the present study in accordance with the reduced migratory capacities and the parameters in the metastasis experiments 9.

Consequently, these findings suggest the need to focus on integrin functions and their restrictions (e.g. in their downstream signaling) after LysoPC treatment.

### LysoPC C18:0 deregulates the communication of SDC‐4 and PKC

The LysoPC‐induced effects to reduce the migratory capacity of endothelial cells 27, 28 have been related to an activation of the PKCδ 36. Upregulation of PKCδ activity phosphorylates SDC‐4, therefore rendering it unable to oligomerize and thus interfering with PKCα recruitment, which is indispensable for cytoskeletal reorganization and focal cell binding processes 37.

**Figure 4:**
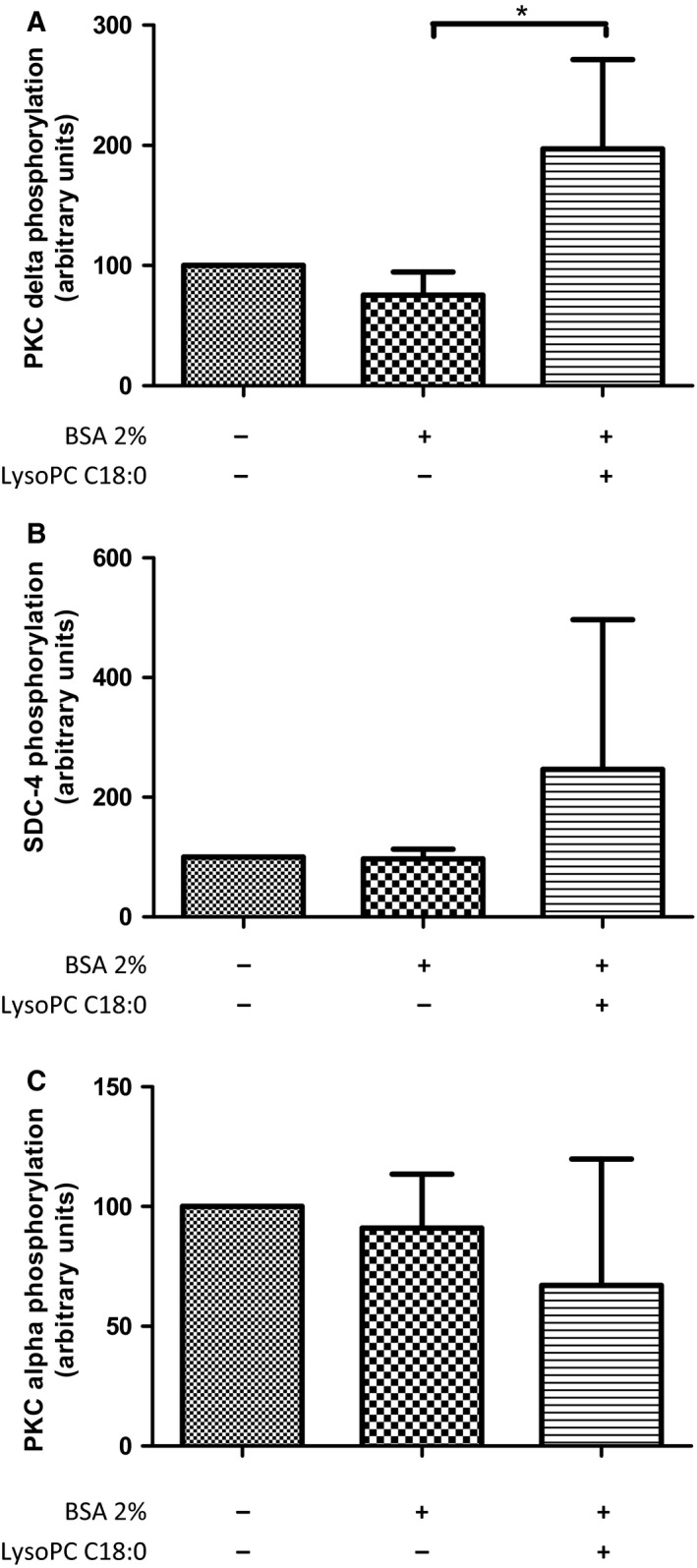
LysoPC deregulates PKC activation. Western blotting to detect the phosphorylation of (A) PKCδ; (B) SDC‐4; and (C) PKCα, in B16.F10 cells dependent on LysoPC C18:0 treatment. Prior to cell lysis, B16.F10 cells were grown without fibronectin in DMEM for 72 h, left untreated or treated with BSA 2% or, additionally, with 450 μm LysoPC C18:0. Normalization of blots before pixel density analysis was performed with StainFree^™^ technology (Bio‐Rad Laboratories GmbH). Data indicate an upregulation of PKCδ activity, consistent with a higher degree of phosphorylated SDC‐4 and a lower activity of PKCα by LysoPC treatment. Asterisks indicate statistical significance (*n* = 3): *P < 0.05; **P < 0.01; ***P < 0.001.

However, these recent findings on PKCδ deregulation by LysoPC 29 differed with respect to experimental context, and particularly in the LysoPC concentrations used (12.5 μm), from the anti‐metastatic approach described in the present study. Nevertheless, to investigate whether LysoPC C18:0 with the effective concentration of 450 μm also induces a similar deregulation in B16.F10 cells, PKCδ activity was checked by western blotting. As indicated in Fig. 4A, 72 h of cell pretreatment with 450 μm LysoPC C18:0 even without fibronectin resulted in a significant increase of PKCδ phosphorylation at the activation loop (Thr507) in contrast to cells that have solely been treated with BSA. Consistent with this increased PKCδ activity, a clear tendency of SDC‐4 phosphorylation at Ser179 could be detected in the LysoPC C18:0 treated cells (Fig. 4B). Following the signaling path further downstream, a slightly reduced phosphorylation at the autophosphorylation site of PKCα (Ser657) becomes evident (Fig. 4C), indicating alleviated activity.

Interestingly, these altered PKC activities are neither associated with, nor induced by changed intracellular calcium levels, as might be expected 38. Intracellular calcium detection by flow cytometry applying calcium green‐1 displayed identical calcium concentrations in B16.F10 cells after LysoPC treatment compared to untreated cells (Fig. S3).

These findings suggest that LysoPC C18:0 not only rigidifies the membrane of B16.F10 cells and thus passively reduces their flexibility but possesses additional properties for deregulating the focal contact formation.

### LysoPC attenuates focal adhesion kinase activity

The formation and controlled disassembly of focal adhesion complexes is an indispensable prerequisite for cell motility and migratory capacity. Amongst multiple components in forming focal adhesion, focal adhesion kinase (FAK) is of key importance with respect to triggering the changes in cell morphology by a phosphorylation of its tyrosine residues 39. Aiming to investigate whether the PKC deregulation by LysoPC C18:0 also affects FAK activity in B16.F10 cells, cellular pretreatment with 450 μm LysoPC C18:0 for 72 h without fibronectin was evaluated by western blotting. In addition, cells pretreated with LysoPC C18:1, which was much less efficient in affecting migration, were also included.

**Figure 5:**
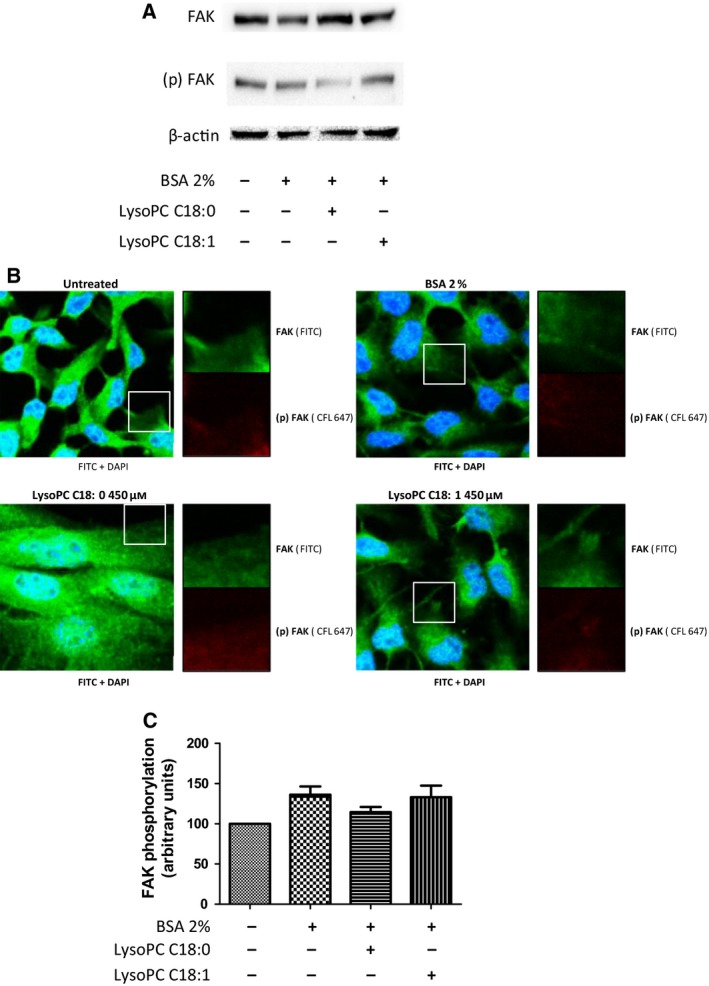
LysoPC C18:0 affects focal adhesion complex formation in B16.F10 cells. (A) Western blotting images of FAK and the Tyr925 phosphorylated (p)FAK in B16.F10 cells as an indicator for focal adhesion complex formation dependent on pretreatment with LysoPC. Prior to cell lysis, the cells were grown without fibronectin and treated with BSA 2%, BSA 2% plus LysoPC C18:0 or LysoPC C18:1 at concentrations of 450 μm or only with DMEM for 72 h. Considering identical levels of FAK, the data indicate a clear reduction of FAK activation in the case of LysoPC C18:0 pretreatment, whereas LysoPC C18:1 apparently leaves the FAK activation unaffected. Representative blots from three independent experiments are shown. (B) Microscopic FAK phosphorylation analysis. Prior to application of antibodies, the cells were cultivated with DMEM for 72 h on fibronectin and treated with BSA 2% or, additionally, with 450 μm LysoPC C18:0 or LysoPC C18:1. Cells were labeled with a fluorescent FAK antibody (large images). Identical areas at the leading edge of cells were selected to focus on activation in the migratory front and labeling of FAK (green) and pFAK (red) was compared. (C) A pixel density analysis was performed comparing the identical zoomed in cell areas at the cell lamellipodia in the FAK and pFAK labeled B16.F10 cells. The ratio of pFAK and FAK intensity is indicated and the lowest value for LysoPC C18:0 pretreated cells is displayed, indicating the lowest FAK activation in line with the blotting data, whereas LysoPC C18:1 obviously is without effect.

Besides observing the general expression levels of this nonreceptor tyrosine kinase, phosphorylation at Tyr925 served as indicator for FAK activation in the process of cell protrusion formation and migration 40. The data generally revealed no clear deviations in FAK protein expression in B16.F10 cells with respect to the treatment regime (Fig. 5A). However, a slightly higher FAK expression is evident for the LysoPC C18:0 treated cells. By contrast, the data clearly indicate that the phosphorylation at Tyr925 is strongly reduced under the influence of LysoPC C18:0. Interestingly, the mono‐unsaturated LysoPC C18:1 did not show this attenuated activation of FAK.

As a further confirmation of reduced FAK activity at the cell functional level, B16.F10 cells were allowed to spread on fibronectin, and FAK activation was visualized microscopically. Therefore, cells were treated with a fluorescent FAK antibody, which labeled both activated and non‐activated forms of FAK, as indicated in green in Fig. 5B. It is evident that LysoPC C18:0 treated cells displayed the highest intensity of labeled FAK, which is in line with the blotting data shown in Fig. 5A. To follow the FAK activation with a focus on migratory capacity at the leading edge of cells, we zoomed in on identical areas at the cellular front and compared the labeling intensity of FAK and phosphorylated FAK, which is indicated in red. A pixel density determination related to those cell front areas considering the ratio of pFAK and FAK, indicating focal complexes with active FAK, were compared in Fig. 5C. The data show that, after pretreatment with 450 μm LysoPC C18:0 for 72 h, cells display a reduction of FAK phosphorylation by approximately 17%. In contrast, LysoPC C18:1 induced no change in FAK activity.

**Figure 6:**
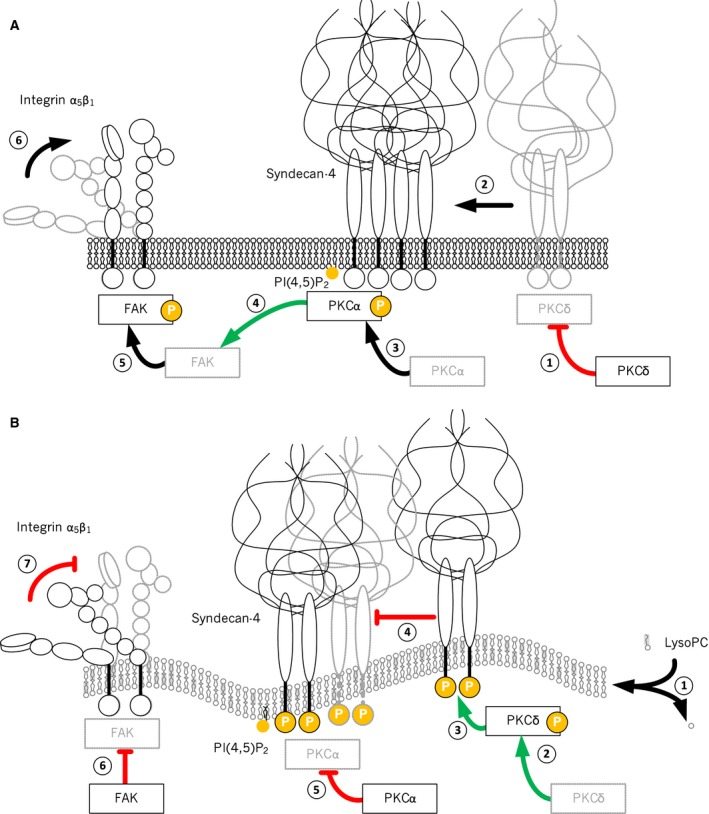
Schematic illustration of the formation of focal adhesion complexes and the postulated interference by LysoPC C18:0. (A) Under physiological conditions, the proteoglycan SDC‐4 tends to oligomerize (2) when contacting extracellular matrix substrates. This triggers the formation of a ternary complex with PIP_2_ and the recruitment of PKCα (3) leading to PKCα activation. This contributes to the formation of focal adhesion complexes (5), which consequently drives integrins into a high affinity binding state (6). (B) LysoPC pretreatment induces a massive incorporation of LysoPC FAs into cell membrane phospholipids (1), which, in the case of LysoPC C18:0, results in membrane rigidification. This leads to an activation of PKCδ (2), which in turn phosphorylates SDC‐4 (3) and impedes their oligomerization (4). Subsequently, the formation of a ternary complex and PKCα activity (5) is reduced, which finally attenuates focal adhesion complex formation (6) and retains integrins in a low affinity binding conformation (7).

These findings indicate a consistent mechanism of saturated LysoPC activity with respect to deregulating cell adhesion, which is illustrated schematically in Fig. 6. The costimulatory function of SDC‐4 in integrin binding, as suggested by Morgan *et al*. 35, implies the formation of a ternary complex of oligomerized SDC‐4 with phosphatidylinositol 4,5‐bisphosphate (PIP_2_) and recruited PKCα 41, which ultimately activates FAK for a cytoskeletal reorganization 42. FAK is essential for the formation of focal contacts containing β_1_ integrins in a high‐affinity binding mode (Fig. 6A). By contrast, the disturbance of lateral membrane order by saturated LysoPC‐derived FAs competes with the ternary complex formation because PKCδ phosphorylates SDC‐4, thus impeding oligomerization and recruitment of PKCα and deregulating the complex machinery of focal contact formation. This finally leaves integrins in a low affinity binding state (Fig. 6B).

## Discussion

In the present study, we demonstrate that saturated LysoPC has a strong impact on focal adhesion formation in melanoma cells, which results in a reduced tumor cell migration. Thus, we provide a functional explanation for the anti‐metastatic effects of LysoPC at higher than the physiological concentrations in plasma that have been reported previously 11. The mode of action of LysoPC activity appears complex, although it is obviously not mediated via activation of GPCRs, at least in the B16.F10 cellular background. The initial trigger of several, engaging events is apparently the massive incorporation of LysoPC derived FAs into the membrane phospholipids. Because there appears to be no preference for specific lipid species and a high tolerance for their membrane incorporation by transacylation 9, FAs biophysically affect the membrane properties in relation to their degree of saturation. Consequently, saturated FAs such as stearic acid (C18:0) mediate a rigidification of the tumor cell membrane, which we could demonstrate by FRAP experiments (B16.F10 cells) or by fluorescence anisotropy using 1‐(4‐trimethylammoniumphenyl)‐6‐phenyl‐1,3,5‐hexatriene *p*‐toluenesulfonate (MV3 human melanoma cells) in previous studies 9, 43.

The impact of a changed membrane fluidity on tumor cell malignancy has hardly been investigated 44, 45 but, in general, an inverse relationship of membrane rigidity and malignancy is proposed. However, the question of whether cell membrane rigidification affects the proliferation or metastasis has not yet been answered. Recently, breast cancer membrane fluidity was shown to accelerate cell adhesion and metastatic capacity 10 and the 3D migration of ovarian cancer cells could be modulated by affecting their stiffness 13. However, adding to this open field, we introduce a potential scenario. We provide evidence that a rigidified membrane deregulates the formation of focal contacts, which are essential for cell migration. The proteoglycan SDC‐4 appears to be of key importance. Rather than inducing membrane rigidification as a consequence of its FA incorporation, LysoPC C18:0 instead induced a phosphorylation of SDC‐4 by PKCδ, which impedes SDC‐4 to form a ternary complex with PIP_2_ and PKCα as a prerequisite for focal complex formation. This process is indispensable for integrin activation and thus explains the attenuated integrin binding capacity in migration upon LysoPC C18:0, as demonstrated both in the present study and previously 11, without changing the expression levels of integrins or SDC‐4. Because only a slight deviation in the nature of the FAs (e.g. the change from C18:0 to C18:1 in LysoPC) leads to a loss in this deregulating activity, the importance of rigidification by saturated FAs is strongly emphasized.

Considering the physiological consequences of these findings, LysoPCs in plasma represent the natural mixture of saturated and unsaturated FAs of all phospholipids. Therefore, an autoregulation of tumor cells driving themselves into an attenuated metastatic capacity by the accumulation and metabolism of saturated species cannot be expected. Furthermore, the LysoPC concentrations used in the present study are slightly higher than the physiological concentrations. In light of potential therapeutic prospects, the controlled application of saturated FAs or saturated LysoPCs does not appear to be an easy and efficient option 9.

By contrast, the spatial feeding of solid tumors with saturated FA or saturated LysoPCs to affect their metastatic capability appears to be an interesting option. This has obviously been achieved as a vital ‘side effect’ of liposomal strategies but is not yet recognized as such in practice. Recently, the application of empty liposomes made from saturated phospholipids in a pancreatic tumor cell model strongly attenuated the number of metastatic foci in the mice lungs, whereas the primary tumors have not been affected by these liposomes 11, 46. In light of the current findings, the ambiguous effect of empty liposomes is quite plausible and at least partly depends on an affected formation of focal contacts in pancreatic tumor cells. Liposomes tend to passively accumulate in solid tumor tissues in relation to the enhanced permeability and retention effect 47. Because pancreatic tumor cells reveal a phospholipase A2 activity, which creates saturated LysoPC in the local tumor cell microenvironment, LysoPC derived FAs are incorporated in tumor cell membranes and thus influence tumor cell adhesion and migration, as shown in the present study. Both capacities are pivotal for tumor cell dissemination. These postulations lead to new considerations with respect to liposomal targeting strategies in cancer therapy.

In the present study, we reveal for the first time that saturated LysoPC affects the formation of focal contacts on tumor cells, which is a fundamental process for cell migration and tumor metastasis. These data shed new light on the field of lipid metabolism of tumor cells and their potential consequences for intracellular signaling pathways as a result of changing the membrane properties.

## Experimental procedures

### Reagents

LysoPC C18:0 (1‐stearoyl‐2‐hydroxy‐sn‐glycero‐3‐phosphocholine) and LysoPC C18:1 (1‐oleoyl‐2‐hydroxy‐sn‐glycero‐3‐phosphocholine) were purchased from Avanti Polar Lipids Inc. (Alabaster, AL, USA). BSA was from Sigma‐Aldrich Chemie GmbH (Steinheim, Germany). All salts and buffers were of analytical grade and were purchased as indicated.

### Cell culture

The murine melanoma cell line B16.F10 was cultivated in Dulbecco's modified Eagle's medium (DMEM) with the addition of 10% (V/V) fetal bovine serum (both from Sigma‐Aldrich Chemie GmbH), 1% (V/V) penicillin/streptomycin solution and 1% (V/V) l‐glutamine solution (both from PAN Biotech GmbH, Aidenbach, Germany) as described previously 11. HEK293 cells were cultured in DMEM supplemented with 10% (V/V) fetal bovine serum, 1% (V/V) penicillin/streptomycin at 37 °C and 5% CO_2_ (Sigma‐Aldrich Chemie GmbH).

Tests for the absence of mycoplasms were performed routinely every month. LysoPC treatment of B16.F10 cells was performed with LysoPC C18:0 or LysoPC C18:1, achieving a final concentration of 450 μm and 2% (m/V) BSA for an incubation time of 72 h.

To prepare lysates of the LysoPC‐treated and untreated B16.F10 cells, cells in sub‐confluent flasks were washed twice with ice‐cold PBS (PAN Biotech GmbH) and incubated with lysis‐buffer and 1 mL of cell extraction buffer (both from Life Technologies GmbH, Darmstadt, Germany) for 10 min. Afterwards, cells were manually detached from the cell culture flasks and the cytosolic fraction was isolated via centrifugation at 17 000 ***g*** for 15 min.

### Western blotting

Using SDS/PAGE, 25 μg of protein for each approach were separated prior to blotting. Precast gels were used differing from 7.5% over 10% to 12.5% in the polymerization degree (Mini‐PROTEAN^®^ TGX^™^ Stain‐Free^™^; Bio‐Rad Laboratories GmbH, Munich, Germany). Proteins were transferred to Roti^®^‐PVDF membrane (Carl Roth GmbH, Karlsruhe, Germany). The membrane was blocked with skimmed milk powder in Tris‐buffered saline‐Tween 20 (with 0.2% Tween 20) for 60 min, followed by three washing cycles of 10 min using Tris‐buffered saline‐Tween 20. Afterwards, membranes were incubated with primary antibodies for a total of 60 min at room temperature and then incubated at 4 °C overnight. Membranes were rinsed again three times before applying the secondary antibodies for 90 min. Primary antibodies were diluted 1 : 200 or 1 : 400; secondary antibodies 1 : 10 000 (both purchased from Santa Cruz Biotechnology Inc., Heidelberg, Germany). After rinsing the secondary antibodies, membranes were detected using Clarity^™^ ECL Western Blotting Substrate (Bio‐Rad Laboratories GmbH). For quantitative determination, the StainFree^™^ technique was employed (Bio‐Rad Laboratories GmbH), as well as normalization, against the housekeeping protein β‐actin, which allows the imaging of whole lysates in SDS/PAGE before blotting and normalization against the total protein. Pixel density analysis was performed with the image lab (Bio‐Rad Laboratories GmbH).

### Label‐free DMR assay

DMR measurements were performed using either the Epic^®^ System (Corning Inc., Tewksbury, MA, USA) or the EnSight^®^ System (PerkinElmer, Waltham, MA, USA) as described previously in detail 32. Briefly, 30 000 B16.F10 or 18 000 HEK293 cells were seeded onto 384‐well biosensor plates in culture medium and incubated overnight at 37 °C and 5% CO_2_. The next day, the cells were washed twice with Hanks’ balanced salt solution (containing 20 mm Hepes) and equilibrated for at least 1 h at 37 °C in the DMR reader. After a baseline read, compounds were added and the resulting DMR was monitored. As positive controls for signaling capability and G protein stimulation, the direct adenylyl cyclase activator forskolin and the pan G protein activator ${\text{AlF}}_{4}^{-}$ as a mixture of AlCl_3_ and NaF (all from Sigma‐Aldrich Chemie GmbH) were used.

### Confocal imaging

Confocal images were taken with a A1R (Nikon GmbH, Düsseldorf, Germany). Cells were seeded on fibronectin (10 μg·mL^−1^; Sigma‐Aldrich Chemie GmbH) coated coverslips (Paul Marienfeld GmbH & Co. KG, Lauda Königshofen, Germany). After the incubation period, cells were washed with PBS, fixated with 3.7% formaldehyde (Merck KGaA, Darmstadt, Germany) in PBS solution and permeabilized with 0.05% Triton X‐100 (Carl Roth GmbH + Co. KG, Karlsruhe, Germany) solution in PBS. Next, unspecific binding sites were blocked with 1% BSA (Sigma‐Aldrich Chemie GmbH) in PBS solution. Primary [FAK rabbit, pFAK (Tyr 925) goat] and secondary (rabbit FITC, goat CFL 647) antibodies were purchased from Santa Cruz Biotechnology Inc. Nuclei were stained with 4′,6‐diamidino‐2‐phenylindole (Sigma‐Aldrich Chemie GmbH). Pixel density measurements were performed with photoshop (Adobe Systems Inc., San Jose, CA, USA).

### Cell migration

Cell migration assays were performed as described previously 43. In total, 5000 B16.F10 cells were seeded on fibronectin coated (10 μg·mL^−1^; Sigma‐Aldrich Chemie GmbH) or uncoated 24‐well plates (STARLAB GmbH, Hamburg, Germany). After 72 h of exposure to the respective assay concentrations, a scratch was conducted with a 200‐μL pipette tip (STARLAB GmbH), medium was removed and fresh medium was added. Wound healing was observed every 30 min for 12 h with an A1R (Nikon GmbH) with 10‐fold magnification at 37 °C and 5% CO_2_. Migration speed was calculated as linear regression of reduced scratch wound over time.

### Determination of LysoPC cytotoxicity by the MTT assay

Potential cytotoxic effects of LysoPC C18:0 and C18:1 in B16.F10 cells were determined by the 3‐(4,5‐dimethylthiazole‐2‐yl)‐2,5‐diphenyltetrazolium bromide (MTT) assay (Sigma‐Aldrich Chemie GmbH) as described previously 48. LysoPC was used in concentrations ranging from 10^−2^ to 10^−8^ m. The total volume in each well of 96‐well plate was 100 μL with 20 000 cells·well^−1^.

### FACS analysis of SDC‐4, GPR4 expression and intracellular calcium

Flow cytometry analysis was performed using a FACS Calibur (Becton Dickinson, Heidelberg, Germany). To determine cellular SDC‐4 and GPR4 expression, primary antibodies were obtained from R&D Systems (Wiesbaden Nordenstadt, Germany), in addition to secondary FITC‐labeled donkey anti‐goat antibodies that were obtained from Santa Cruz Biotechnology Inc.

To determine intracellular Ca^2+^ concentrations, 150 000 B16.F10 cells were incubated with 1.2 μm calcium‐green‐1 (Life Technologies GmbH) solution at 37 °C for 30 min. After washing the cells twice with PBS, fluorescence was quantified by flow cytometry.

### Statistical analysis

Analysis of variance was used for statistical analysis (*P < 0.05; **P < 0.01; ***P < 0.001). Samples were generally measured in triplicates if not indicated otherwise.

## Author contributions

TR and BJ performed the experiments. MG designed and performed the EPIC measurements. EK assisted with data interpretation. UM designed experiments and co‐wrote the paper. MS and GB designed the experiments and wrote the paper.

## Supporting information


**Fig. S1.** Investigation of GPCR‐mediated effects of LysoPC.
**Fig. S2.** Expression of syndecan‐4 in B16.F10 cells with respect to LysoPC pretreatment.
**Fig. S3.** Steady‐state calcium levels of B16.F10 cells under the influence of LysoPC.
