# Supplementary material for: The molecular mechanism by which saturated lysophosphatidylcholine attenuates the metastatic capacity of melanoma cells
Source: FEBS Open Bio. 2016 Nov 24;6(12):1297–309. doi: 10.1002/2211-5463.12152 (PMC5324772; doi:10.1002/2211-5463.12152)
Supplement: Supplementary file 1 — Fig. S1. Investigation of GPCR‐mediated effects of LysoPC. Fig. S2. Expression of syndecan‐4 in B16.F10 cells with respect to LysoPC pretreatment. Fig. S3. Steady‐state calcium levels of B16.F10 cells under the influence of LysoPC. [file FEB4-6-1297-s001.pdf]

Suppl. Fig. 1

A

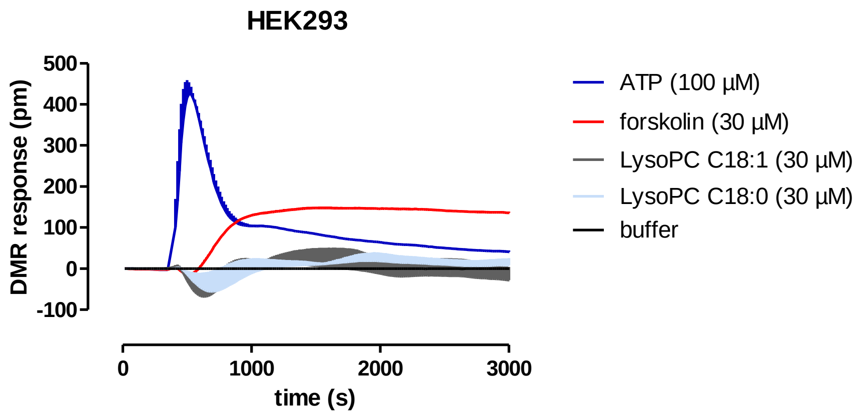

B

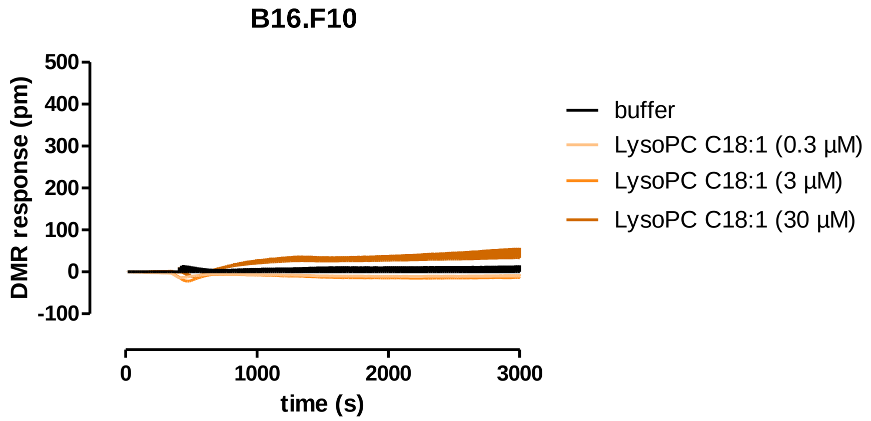

Suppl. Fig. 2

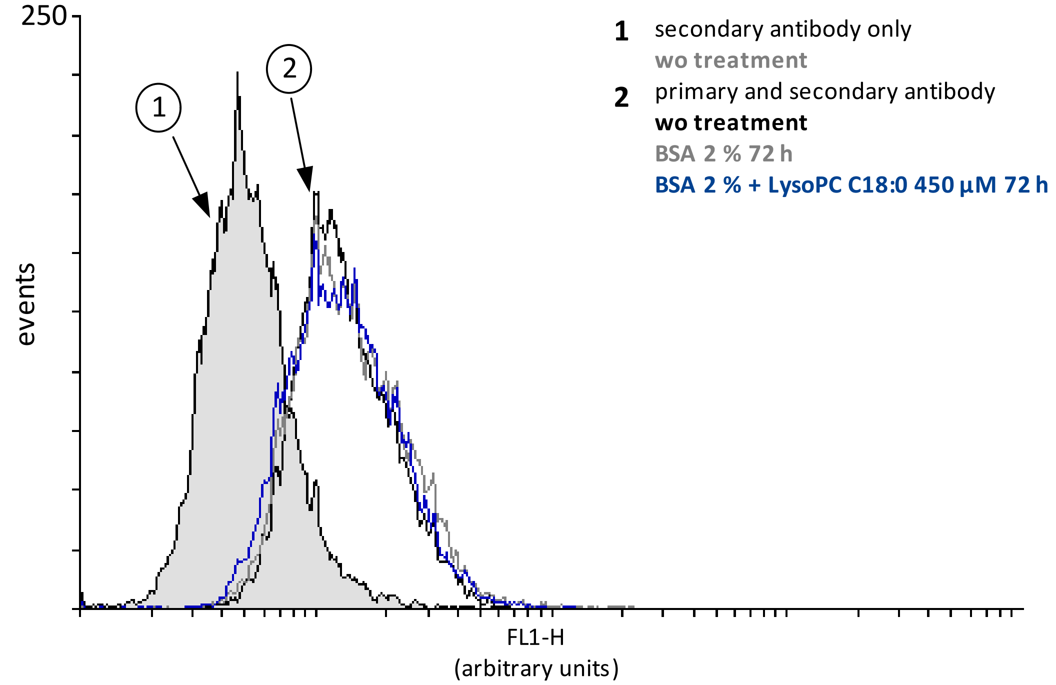
250
events
FL1-H
(arbitrary units)
1
2
1 secondary antibody only
wo treatment
2 primary and secondary antibody
wo treatment
BSA 2 % 72 h
BSA 2 % + LysoPC C18:0 450 µM 72 h

Suppl. Fig. 3

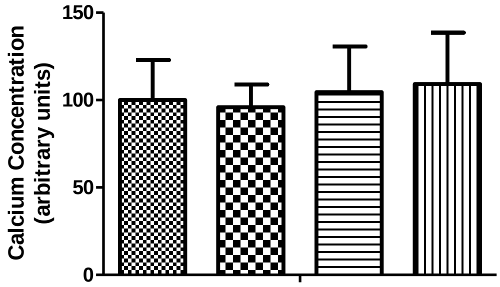

BSA 2 %	-	+	+	+
LysoPC C18:0	-	-	+	-
LysoPC C18:1	-	-	-	+

Legends to supplementary Figures:

Supplementary Figure 1: Investigation of G protein-coupled receptor-mediated effects of LysoPC

A: DMR traces of HEK293 cells stimulated with ATP (100 µM), forskolin (30 µM), LysoPC C18:1 (30 µM) and LysoPC C18:0 (30 µM) (n=3).

B: DMR recordings of different LysoPC C18:1 concentrations acting on B16.F10 cells. Shown are mean values + sem (n=3).

Supplementary Figure 2: Expression of Syndecan-4 in B16.F10 cells with respect to LysoPC pretreatment

Flow cytometric measurements are displayed as histograms. Peak 1 shows control fluorescence levels of cells with secondary FITC labeled antibody in absence of a SDC-4 specific antibody. Peak 2 presents the fluorescence level in presence of primary and secondary antibody. The black line shows control B16.F10 cells without further treatment, the grey line represents cells treated with 2 % BSA containing medium for 72 h and the blue line displays cells treated with 2 % BSA and 450 µM LysoPC C18:0 for 72 h.

Supplementary Figure 3: Steady-state Calcium levels of B16.F10 cells under the influence of LysoPC

Using the fluorescent probe Calcium-Green-1, Calcium levels were measured by flow cytometry. Cells were completely untreated, or treated for 72 h with BSA 2 % alone or with either 450 µM LysoPC C18:0 or LysoPC C18:1 (n=8)
